# An Intracellular Antioxidant Determines the Expression of a Melanin-Based Signal in a Bird

**DOI:** 10.1371/journal.pone.0003335

**Published:** 2008-10-03

**Authors:** Ismael Galván, Carlos Alonso-Alvarez

**Affiliations:** 1 Department of Evolutionary Ecology of the Museo Nacional de Ciencias Naturales (CSIC) in Madrid, Madrid, Spain; 2 Instituto de Investigación en Recursos Cinegéticos, IREC (CSIC, UCLM, JCCM) in Ciudad Real, Ciudad Real, Spain; Georgia State University, United States of America

## Abstract

To understand how traits used in animal communication evolved and are maintained as honest signals, we need to understand the mechanisms that prevent cheating. It has been proposed that honest signaling is guaranteed by the costs associated with the signal expression. However, the nature of these costs is still under debate. Melanin-based signals are intriguing because their expression seems to be tightly controlled by genes and the resource involved (i.e. melanin) seems to be not limited. However, in vertebrates, low levels of a key intracellular antioxidant (i.e. glutathione) are needed to promote melanogenesis. We propose that melanin-based ornaments can signal the ability to cope with oxidative stress because those individuals with low enough levels of glutathione, such as those required for melanin production, should manage well the whole of the antioxidant machinery in order to maintain a certain oxidative status. We analysed the expression of a melanin-based signal: the well-known black stripe of the great tit (*Parus major*). Great tit nestlings were injected with a specific inhibitor of glutathione production (DL-buthionine-*S*,*R*-sulfoximine; BSO) throughout their development. BSO effectively decreased intracellular glutathione levels without apparent side effects on growth or body condition. Instead, treated nestlings developed black breast stripes 70–100% larger than controls. Moreover, treated nestlings also compensated the decrease in glutathione levels by increasing the levels of circulating antioxidants. Results indicate that melanin-based signals can be at least partially permeable to environmental influences such as those associated to oxidative stress. They also reveal a potential handicap associated to the expression of this kind of signals. Finally, although other contributing factors could have been present, our findings emphasize the role of oxidative stress in shaping the evolution of animal signals in general and, in particular, those produced by pigments.

## Introduction

The evolution of signals used in animal communication is often explained by the honesty in the information content of these traits [1]. In this way, honesty has become to be one of the main issues in signaling theory. The “handicap principle”, first proposed by Zahavi [2], states that communication is essentially honest, and this honesty is maintained by the cost of the signal. This ensures that the investment in producing the signal is acceptable to an honest signaler but prohibitive to a cheater. However, the nature of the cost of handicap signals is still under debate.

Among potential costs associated to signaling, the allocation of limited resources in the construction of the signaling trait has been often suggested, especially in order to explain the evolution of colorful signals. These particular traits are mainly produced by two different pigment families: carotenoids (yellow-red traits) and melanins (mostly brown-black traits) e.g. [3]–[5]. We know that carotenoids are scarce in the environment and exclusively obtained from diet [4]. They have also immuno-stimulant and antioxidant properties [4]. In this way, it has been proposed that those individuals fully expressing carotenoid-produced traits could signal their foraging skills [4] and/or their quality in managing the trade-off between signal expression and self-maintenance [6].

Meanwhile, the costs derived from the production of melanin-dependent traits are less evident [7], [8]. Melanins are *de novo* synthesized by the organism from phenylalanine and tyrosine amino acids [9]. Thus, we could assume that melanins would be not a limited resource for trait building such as carotenoids. Furthermore, it has been argued that the expression of carotenoid-dependent traits is very sensitive to environmental factors, whereas melanin-based traits are strictly controlled by genes e.g. [10], [11]. However, several experiments have demonstrated that adverse rearing conditions, parasite infections and diet quality influence the expression of different melanin-based signals [8], [12]. Therefore, they could express a certain phenotypic plasticity [13], [14].

Several hypotheses have been proposed to explain the cost of melanin-based signals. Some of them suggest that melanin-based ornaments are signals of foraging skills such as firstly suggested for carotenoid-dependent traits. Experiments with captive birds indicate that the availability of calcium [15]; see also [16], phenylalanine and tyrosine [17] could be limiting for the production of melanins see also [18]. Meanwhile, other hypotheses propose that the cost is related to those hormones involved in melanogenesis. Thus, both testosterone, often linked to the production of sexually-dimorphic melanic traits [7], [19]; but see [20], and melanocyte-stimulating hormone (MSH) promote immunosuppression [21]–[23]. The expression of melanin-based traits should be hence costly in terms of higher susceptibility to infections [5], [22], [24].

However, the role of oxidative stress, that is, the imbalance between production of reactive oxygen species (ROS) and availability of antioxidant compounds [25], has been barely addressed in the study of melanic signals. Oxidation is a ubiquitous phenomenon intimately related to life on earth, and hence, should shape the expression of most phenotypes [25]. In this sense, oxidative stress would have influenced the evolution of carotenoid-dependent traits because carotenoids are sensitive to oxidation and can act as antioxidants [4], [6], [26]. Similarly, melanins could behave as antioxidants and melanocyte development is very sensitive to oxidative stress [5], [24]. Thus, it has been recently proposed that the proper development of melanin-based traits could signal the performance of antioxidants in the integuments i.e. [5], [24].

**Figure 1:**
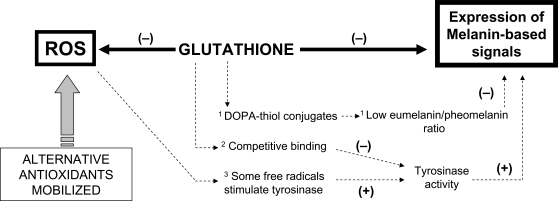
Hypothesized mechanism at the basis of the cost of melanin-based signals. High glutathione levels simultaneously neutralize reactive oxygen species (ROS) and inhibit melanin production. Individuals fully expressing melanin-based signals pay the cost of a decrease in their antioxidant power. The information content of the signal reflects the capacity to mobilize alternative antioxidant systems. Different mechanisms have been proposed to explain the inhibition of melanisation by glutathione: 1. Reaction of thiol groups of glutathione with L-dopaquinone to form dopa-thiol conjugates, which are precursors of phaeomelanin, leading to an apparent inhibition of pigmentation due to the lighter color of phaeomelanin [67], [68]. 2: Interaction of thiol groups with the tyrosinase active site, inhibiting tyrosinase hydroxylation [67]. Tyrosinase is the main catalytic agent for melanin production in vertebrates [69]. 3: Depletion of free radicals and peroxides by glutathione, as some of these agents stimulate tyrosinase activity [70].

Melanin has also an overlooked property that could definitively link its production to oxidative stress: it depends on glutathione levels. Glutathione (GSH) is a tripeptide thiol found in virtually all animal cells, functioning in the reduction of the disulfide linkages of proteins, in the synthesis of the deoxyribonucleotide precursors of DNA and in the protection of cells against free radicals [27]. With respect to this last function, GSH is often considered as the most important intracellular antioxidant e.g., [28], [29]. Interestingly, GSH also serves as an agent regulating the process of melanogenesis. Low GSH levels have been related to the deposition of melanin in the skin of humans and other mammals [30], [31], whereas high GSH levels inhibit melanogenesis (Figure 1).

Here, we hypothesize that melanin-based ornaments signal the ability to cope with oxidative stress because those individuals with low enough levels of glutathione, such as those required for melanogenesis, must have sufficient alternative antioxidant resources to counteract the decrease of GSH levels (Figure 1). Therefore, we propose that melanin production for signalling implies a cost and promotes a handicap. We tested the hypothesis in the great tit (*Parus major*), a passerine bird that displays a well known melanin-based signal, i.e. the black breast stripe. This trait serves as a badge of status in both sexes, larger stripes leading to higher success in agonistic interactions between conspecifics and better access to resources e.g. [32]–[34], as well as providing a mating advantage to males [35], [36]. In this study, we experimentally decreased erythrocyte glutathione levels of great tits during their early development as nestlings by means of repeated injections with a specific glutathione inhibitor (DL-buthionine-*S*,*R*-sulfoximine; BSO). We predicted that treated birds would produce larger stripes, but also mobilize other antioxidants to plasma in order to avoid oxidative stress. Developmental conditions have shown to exert a strong influence on the life history trajectory of vertebrates in general [13], [37], and in birds in particular [38]. Thus, we assume that the result of this manipulation will determine the adult physiology and phenotype, and lastly, lifetime fitness.

## Results

The manipulation did not significantly affect growth or body condition of birds. Body mass gain was not affected by the treatment (*F*_3,169_ = 1.03, *P* = 0.382), though males gained more mass than females (11.02±0.19 g and 10.57±0.19 g, respectively; *F*_1,177_ = 16.09, P<0.0001; initial body mass: *F*_1,197_ = 130.01, *P*<0.0001). Similarly, body condition (i.e. size-corrected body mass) of 15 days old birds was not affected by the treatment (*F*_3,171_ = 0.97, *P* = 0.407; tarsus length: *F*_1,187_ = 42.34, *P*<0.0001). Neither body mass nor tarsus length at 15 d old differed between treatments when they were separately tested (*F*_3,171_ = 0.71, *P* = 0.546 and *F*_3,171_ = 1.03, *P* = 0.379, respectively). Only the sex factor remained in both models (both *P*<0.001) because males were heavier and larger than females (males: 18.28±0.199 g and 19.74±0.109 mm; females: 17.78±0.199 g and 19.16±0.109 mm; for mass and tarsus length, respectively). The rest of covariates and interactions in all these models (see Methods) were removed as non-significant terms. Initial body mass did not differ between treatments nor was affected by any factor or covariate (all *P*-values>0.05).

**Figure 2:**
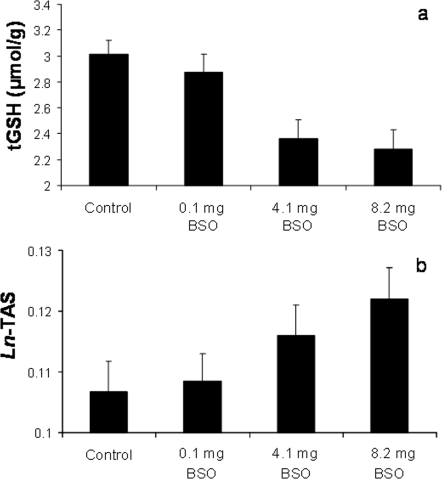
Effect of a glutathione inhibitor on the antioxidant machinery of great tits. Nestlings treated with different doses of a glutathione inhibitor (DL-buthionine-*S*,*R*-sulfoximine; BSO) during development presented lower total glutathione levels in erythrocytes (*A*) and higher total antioxidant status (TAS) in blood plasma (*B*). TAS values were *Ln*-transformed to meet the normality assumption. Least squared means±SE from mixed models including the nest identity as a random factor.

The treatment successfully decreased total glutathione (tGSH) levels in erythrocytes (*F*_3,170_ = 28.3, *P*<0.0001; Fig. 2A), showing an ordered pattern regard to the treatment doses (Fig. 2A). Meanwhile, the levels of plasma antioxidants (i.e. total antioxidant status; TAS) showed the reverse pattern, increasing in those birds treated with higher BSO doses (*F*_3,130_ = 2.84, *P* = 0.042; Fig. 2B). The sex, all the covariates and their interactions were removed as non-significant terms in both tGSH and TAS models.

**Figure 3:**
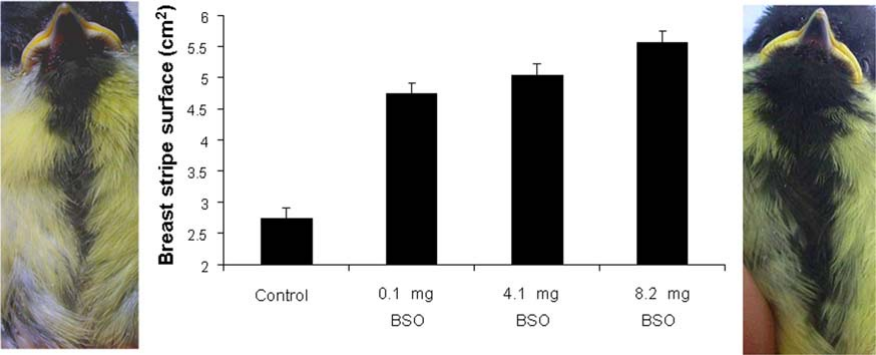
The reduction in glutathione levels was translated into signal expression. Great tit nestlings treated with different doses of a glutathione inhibitor (DL-buthionine-*S*,*R*-sulfoximine, BSO) throughout their development showed larger breast stripes. Least squared means±SE from mixed models including the nest identity as a random factor. Pictures on left and right sides of the figure illustrate a control and a highest BSO-dose nestling.

Finally, the surface of the breast stripe strongly increased with the BSO treatment (*F*_3,163_ = 91.56, *P*<0.0001; Fig. 3). The body mass measured at the beginning of the experiment (i.e. 5 days old) and the sex of the chick were retained in the model. Thus, heavier birds showed larger stripes (*F*_1,175_ = 10.42, *P* = 0.0011; slope±SE = 0.205±0.063), and males showed larger stripes than females (*F*_1,169_ = 12.07, *P* = 0.0007; 4.76±0.18 cm^2^ and 4.28±0.18 cm^2^, for males and females, respectively). No other covariate influenced this result.

## Discussion

The experimental treatment had success in manipulating erythrocyte glutathione levels in great tits and also dramatically increased the surface of the black breast stripe (70–100% increase; Fig. 3). The range of tGSH values at the highest BSO dose was within the range showed by control birds (1.29–3.75 and 0.85–4.64 µmol/mL, respectively), which suggests that our procedure did not produce artificial phenotypes in terms of glutathione. Moreover, the body condition of nestlings did not significantly differ among treatments, suggesting that there were not relevant side effects of BSO. Nonetheless, we must be cautious in accepting any null hypothesis because our statistical power was in fact low (range: 0.27–0.36; calculated from 39).

**Figure 4:**
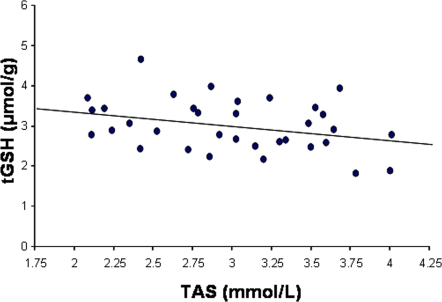
The level of total glutathione (tGSH) in erythrocytes was negatively related to the circulating levels of antioxidants (i.e. total antioxidant status; TAS) in control birds. Dots represent real data, whereas the linear adjust was obtained from the model including the nest identity as random factor.

On the other hand, the amount of antioxidants in plasma (TAS) increased, supporting our hypothesis that glutathione depletion implies a cost in terms of increased antioxidant mobilization to compensate for the GSH scarcity (Figure 1). Such a cost would maintain the honesty of the breast stripe as a signal of individual quality [2]. In this way, if individuals with high enough level of antioxidants can afford the decrease of GSH that allows melanization, we would expect a negative correlation between both parameters. Indeed, such a correlation was present in controls (*F*_1, 25.8_ = 4.77, *P* = 0.039; slope±SE −8.01±3.67, Fig. 4). However, a formal demonstration of the handicap mechanism we are proposing here, would require the observation that —after choosing any measure of individual quality— low quality individuals are unable to increase both the expression of the signal and the level of alternative antioxidants after a decrease in glutathione levels to the same degree than high quality individuals [1]. Alternatively, low quality individuals could be still able to signal without compensating the glutathione decline, but they would pay the effort latter in life (e.g. in terms of accelerated aging). Nonetheless, if the capacity to allocate antioxidant resources for compensating the low levels of GSH does not represent the base of a handicap, GSH-mediated melanin-based coloration would still act as a cue of individual quality, with highly expressed melanic traits indicating that oxidative stress is efficiently dealt with by the cell metabolism (e.g. a better respiratory efficiency reducing ROS production; e.g. 40).

If we assume that glutathione depletion is costly and assures the honesty of the signal, it must be noted here that the nature of the cost we are proposing differs from other costs previously suggested for assuring the honesty of melanin-based signals. In our case, the key element (i.e. glutathione) is not directly involved in building the signal, but acts as a modulating agent in their expression. In contrast, the melanin molecule or melanin precursors (i.e. phenylalanine and tyrosine amino acids) could act as antioxidants or affect homeostasis [24], [41], which would imply the existence of trade-offs in the allocation of these resources between the trait building and the antioxidant defense.

In any case, a cost mediated by glutathione suggests that melanin-based traits are in fact signals of the individual capacity to manage oxidative stress [5], [24]. Nonetheless, we must underline that this is a complex system and the proposed pathway (Figure 1), although relevant, could only represent a part of the complete mechanism. It is likely that melanin production is constrained by a variety of factors rather than by a single one [5]. The fact that the expression of melanin-based signals could also depend on the state of different functions of the organism (i.e. immuno-competence, nutrition and oxidative status) suggests that these traits should depend on many different loci, and therefore provide a large mutational target that would be resistant to genetic erosion, such as predicted under the pressure of sexual selection [42].

In this sense, other alternatives proposed to explain the costs associated to these traits can be, at least partially, explored in our dataset. As previously commented, some of them suggest that the production of melanins can be constrained by the presence of melanin precursors in the diet (i.e. amino acids and calcium) [15], [17]. Here, a positive correlation between breast stripe surface and body condition (residuals of body mass on tarsus length) was detected in 15 d old control birds (*F*_1,50.9_ = 7.76, *P* = 0.008; slope±SE = 0.243±0.087), which could indicate a better access to food and hence to these particular resources see also [18]. On the other hand, we can also explore the hypothesis that melanin-based signals are costly due to their dependence on testosterone levels, at least in those species with sexually dimorphic melanic traits [22], but see [20]. Since testosterone increase metabolic rates in birds [43], which may imply increased free radical production [25], we could expect that higher hormone levels in males could have increased the susceptibility to oxidative stress see [44]. In great tits, male chicks show higher testosterone levels than females during first days of life [45] and we here show that 15d old male nestlings already present larger breast stripes than females (such as reported in adults) [46]. However, the influence of the sex on tGSH and TAS was not significant, and hence, the interaction between both testosterone- and glutathione-mediated mechanisms cannot be inferred. Nonetheless, we must also consider that although female nestling great tits present lower testosterone levels than males [45], low testosterone titles have still potential to produce some cost, reducing the differences between sexes.

Our results also support the idea that the expression of the melanin-based phenotypes is not so tightly controlled by genes, showing plasticity when exposed to changes in the internal milieu of the organism. In this sense, although GSH metabolism is undoubtedly controlled by genes e.g. [47], environmental influences on GSH production has been broadly reported e.g. [48], [49]. Moreover, the fact that our manipulation was carried out during early development could have amplified the effects, determining the life history of individuals [37], [38]. The breast stripe of the great tit is a well known signal of dominance in conflicts for food in winter flocks [32], [34], [50]; but see [33] and during nest defense in the breeding season [35], [36]. Since sexual dimorphism in the trait was present in our nestlings, and since the surface of the stripe strongly correlates between consecutive molts in this species (*r* = 0.91, in ref. 51), our results suggest that the effects of the manipulation should extend to adult phenotypes and fitness. Nonetheless, comparable experiments in adults are still needed to corroborate the presence of the proposed mechanism throughout adulthood.

Oxidation is a ubiquitous phenomenon intimately related to life on earth. In view of the fact that the production of many yellow-orange-red signals is based on carotenoids, which are also antioxidants [6], [52] or are susceptible to oxidation [26], our results emphasize the potential role of oxidative stress in shape the evolution of sexual signals in general and, in particular, those produced by pigments. Furthermore, given that melanin is the most common pigment in vertebrate tissues, with functions ranging from antioxidant activity to protection from radiation and mechanical wearing [5], [9], [53], the importance of this overlooked physiological cost for understanding variation in the expression of any melanic phenotypic trait, including human skin [54], seems evident.

## Materials and Methods

### Experimental design

The study was carried out in May–July 2007 in a deciduous forest of Pyrenean oak *Quercus pyrenaica* in Miraflores de la Sierra, Sierra de Guadarrama, Comunidad de Madrid (40° 49′ N, 03° 46′ W, 1352 m a.s.l.). We injected BSO (Sigma, St. Louis, Missouri, USA; product code: B2640) subcutaneously in the back of nestling great tits. BSO is a selective inhibitor of γ-GCS, a key enzyme in the GSH biosynthetic pathway, and it is frequently used to decrease GSH levels in mammals [55]–[58]. It has been also proved to decrease GSH levels in domestic fowl chicks (*Gallus gallus domesticus*) under laboratory conditions [59]. The high specificity of BSO makes this a non-toxic compound that produces no effects different from a decrease in GSH levels [56]. The Spanish agency in charge of environmental policy and animal welfare of Madrid (Consejería de Medio Ambiente, Comunidad de Madrid) approved the manipulation of birds.

201 great tit nestlings from 26 nests were used. BSO was administered to nestlings in 0.1 ml of distilled water at low (1 mg/ml), medium (41 mg/ml) or high (81 mg/ml) doses. The doses were chosen from available data in experiments carried on poultry [59], but also trying to cover a large range of variation. Control birds only received the same volume of distilled water. Samples sizes for each treatment were 53, 51, 55, and 45, for control, low dose, medium dose and high dose birds, respectively. The nestlings were assigned to these treatments following the order of body weights but changing the start of the sequence of treatments in each nest so that all positions in the sequence of weights received the same number of the different treatments. The treatments were administered to nestlings at days 5, 7, 9 and 11 after hatching. On day 11, 150 µl of blood were collected from the brachial vein and immediately stored at 4°C. Blood samples were later centrifuged (within 5 hours) and the cell and plasma fractions were stored separately in liquid nitrogen until biochemical analyses were performed. Two blood samples were rejected for tGSH and TAS analyses due to hemolysis.

On day 15, the nestlings were weighed (accuracy 0.1 g), their tarsus length measured (accuracy 0.01 mm) and a digital photograph of the breast in a standardized position was taken with a graph paper as a reference. The assessment of the melanized breast stripe surface by means of Adobe Photoshop CS was calculated from those photographs by selecting the entire black area of the plumage of nestlings from the throat to the belly, as this method ensures a high repeatability in the particular case of great tits [60]. In our study site, nestlings are almost completely fledged and about to leave the nest at 15 days old [61]. No died nestlings were found during the course of the experiment.

### Total Glutathione

tGSH levels in red blood cells were determined by following the method described by Tietze [62] and Griffith [63] with some particular modifications. Briefly, the blood pellet was thawed and the red blood cells were pipetted by avoiding the pellet surface (i.e. the buffy coat containing white blood cells). Erythrocytes were immediately diluted (1∶10 w/v) and homogenized in a stock buffer (0.01 M PBS and 0.02 M EDTA), always working on ice to avoid oxidation. Three working solutions were made up in the same stock buffer as follows: (I) 0.3 mM NADPH, (II) 6 mM DTNB, and (III) 50 Units of glutathione reductase/mL. An aliquot (0.5 mL) of homogenate of blood cells was vortexed with 0.5 mL of diluted tricholoroacetic acid (10% in H_2_0) three times during 5 seconds each bout within a 15 min period. In the mean time, samples were removed from light and refrigerated to prevent oxidation. Afterwards, the mixture was centrifuged (1125G, 15 min, 6°C) and the supernatant removed. Next steps were carried out in an automated spectrophotometer (A25-Autoanalyzer; Biosystems SA, Barcelona). Solution I and II were mixed at 7∶1 volume, respectively. 160 µL of this new mixture was automatically added to 40 µL of sample (supernatant, see above) in a cuvette. Afterwards, 20 µL of solution III was added after 15 seconds and the absorbance at 405 nm was monitored after 30 and 60 seconds. The change in absorbance was used to determine the total glutathione concentration in red blood cells by comparing the output with the results from a standard curve generated by serial dilution of glutathione from 1 mM to 0.031 mM. Results are given in mmol per gram of pellet. All samples from the same nest were measured in a single assay. Repeatability of this technique [64] was determined on a sub-sample measured twice (*r* = 0.85, n = 20, *P* = 0.002).

### Total Antioxidant Status

TAS of plasma was assessed by means of commercial kits (Randox Laboratories Ltd, Crumlin, UK) adapted to an automated spectrophotometer (A25-Autoanalyzer; Biosystems SA, Barcelona). Briefly, plasma samples were incubated during 15 s with a chromogen composed by metmyoglobin and ((2,2-azino-di-[3-ethylbenzthiazoline sulphonate]); ABTS®). Then, hydrogen peroxide (H_2_O_2_) was added and the sample was incubated during 195 seconds. H_2_O_2_ addition induces the production of the radical cation ABTS, which generates a blue-green color. Color is measured at 600 nm before and after H_2_O_2_ addition, thus determining the change in color. Antioxidants in the plasma sample cause suppression of this color change to a degree which is proportional to their concentration. Results are given as mmol/L of total antioxidants. Unfortunately, some random samples (i.e. regard to plasma volume, nest identity or treatment) were incorrectly captured by the automated pipetting system, exhausting the plasma for new analyses. Thus, TAS could be finally determined on a sub-sample of 150 birds from 25 nests. All samples from the same nest were measured in the same assay. Repeatability [64] was determined on a sub-sample measured twice (*r* = 0.92, n = 30, *P*<0.001).

### Molecular sexing

In order to avoid any potential bias (see Statistical analyses) due to the fact that the breast stripe of the great tit is a sexually dimorphic trait [47], nestlings were molecularly sexed from a sub-sample of the blood cell fraction. DNA from the sex chromosomes (Z and W) was amplified by PCR using the primers P2 and P8 and the procedure described in Griffiths et al. [65].

### Statistical analyses

The impact of the treatment (i.e. a four level fixed factor) on the breast stripe surface, tGSH and TAS levels as well as on body mass and tarsus length (dependent variables) was tested by generalized linear models (PROC MIXED in SAS Software) [39], adding the nest identity as a random factor (always *P*<0.05). Body mass gain was tested by adding initial body mass (5 d old) as a covariate in a model testing body mass increase (15 d minus 5 d mass). Body condition was tested by adding tarsus length to a model analyzing body mass at 15 d old as dependent variable. The sex of the bird (fixed factor) and the number of chicks per nest (covariate) were also added to the models. Body mass and tarsus length (15 d old), body condition (residuals of a regression between body mass and tarsus length at 15 d of age) and initial body mass (5 days old) were also tested as covariates in models analyzing variability in breast-stripe surface, tGSH and TAS levels. We tested all potential models including two-order interactions and following the lowest Akaike Information Criterion (AIC) to obtain the best fitted one [66]. Only significant terms were maintained in the models (*P*<0.05).

TAS levels were *Ln*-transformed to meet the normality requirement. Satterthwaite correction was used to approximate degrees of freedom [39]. Least square means and SE were determined from the models.
